# The first identification of cronstedtite in Cu–Ni–PGE ores of the Talnakh intrusion

**DOI:** 10.1038/s41598-023-49953-x

**Published:** 2023-12-17

**Authors:** T. Yu. Yakich, E. S. Zhimuleva, M. A. Rudmin, A. S. Ruban, P. N. Maximov, M. V. Shaldybin

**Affiliations:** Division for Geology, School of Earth Sciences & Engineering, Tomsk Polytechnic University, Soviet Street, Tomsk, Russia 634050

**Keywords:** Solid Earth sciences, Mineralogy

## Abstract

We present new mineralogical data of cronstedtite from the Southern-2 orebody, located in the South-Western branch of the Talnakh intrusion (Noril’sk area) composed of massive sulfides in which the total amount of oxides and silicates does not exceed 1–3 vol%. The petrographic and mineralogical features of these ores indicated occurrence of fine-grained, fibrous needle like clusters < 50-µm-sized grains of cronstedtite (7.09 Å along its *c*-axis). This mineral confirmed by a number of analytical techniques (powder X-ray diffraction of balk samples, transmission electron microscopy, scanning electron microscopy, Raman and Infrared spectroscopy). Cronstedtite sporadically contains signals of Al, Ni, Ca and filling the cracks and cavities between sulfides of copper (chalcopyrite) and iron (pyrrhotite, pentlandite). In some cases, cronstedtite contains micron-sized PGM, and associates with magnetite. According the X-ray diffraction analysis of the bulk massive ores besides cronstendtite are established kaolinite, gypsum, calcite, quartz, and cristobalite. The findings of cronstedtite in Noril’sk area have never been mentioned publicly before. Its occurrence is the northernmost known locality in the world. Our results imply that the formation of cronstedtite in the Talnakh intrusion could be possible by the active participation low-temperatures fluids within the relatively near-surface (< 2 km of paleosurface) conditions of intrusion emplacement, in contrast to other deep-seated supergiant Cu–Ni–PGE deposits in the world. The conditions of formation in isolated cavities in fresh pyrrhotite-pentlandite-chalcopyrite massive ores of deep level of the Talnakh intrusion could be favorable for the formation of cronstendtite.

## Introduction

The group of Noril’sk Cu–Ni–PGE sulfide deposits are of unique economic interest globally as they contain significant amount of the world’s reserves of the platinum-group elements (PGE), particularly Pd^1–4^. Norilʼsk ranks second after Sudbury in Ni reserves and second after Bushveld in PGE reserves^5,6^. Although many intrusions in the Noril’sk area contain Cu–Ni sulfides, only three of them (Talnakh, Khaeralakh, and Noril’sk 1), which are temporally associated with Permian–Triassic basaltic eruptions^7–9^, contain economic concentrations of strategic and critical metals, such as Cu, Ni, and PGE. The genesis of the Noril’sk deposits has been discussed for several decades. One of the important features of these deposits is their extra-ordinary mineral composition which is important for determining of the formation conditions. The Talnakh deposit (together with the Oktyabr’sky deposit) is characterized by a huge mineralogical diversity compared to other magmatic deposits. The predominant number of modern publications are devoted to platinum group minerals^6,10,11^. Other groups of mineral species have been studied to a much lesser extent^12^. Therefore, new data on rare phyllosilicate, which sensitive to physico-chemical conditions of formation is relevant.

Previously, in the literature devoted to the study of the mineralogy of the Norilʼsk region, there was no mention of the mineral cronstedtite, despite the huge history of studying mineralogy of this region since 1960 year. It is possible that the reason is the fact that cronstedtite is easily confused with ferruginous chlorites, if they are not deeply embedded in the study of the structure of the mineral using special methods (Raman, XRD, TEM, etc.), and given the small size of crystals of 30–50 microns, this should be done problematic enough. This finding and identification may be interested because its occurrence is the northernmost known locality in the world today, and it was discovered in the deposit that is unique in its nature and Cu–Ni–PGE reserves. Cronstendtite as the iron hydrous layer silicate is unique in having ferric iron substituted for silicon in positions of tetrahedral co-ordination. It attracted our attention because we found that it exhibits close intergrowths with palladium minerals and is quite rare mentioned in Cu–Ni–PGE deposits. Cronstedtite is an uncommon mineral that occurs as a hydrothermal product in ore veins in localities in the Czech Republic, Romania, Germany, France, England, the United States, Mexico, Brazil, and Bolivia in such localities as Nagybörzsöny gold ore deposit (Hungary)^13^, Gernrode, Lutherstadt Eisleben, Saxony-Anhalt (Germany)^14^, Lostwithiel, Wheal Maudlin, Wheal Jane (Cornwall, UK)^15,16^, Herja, Chiuzbaia (Romania)^17^ and conversely widespread in carbonaceous chondrites and extraterrestrial bodies, including some dark regions of Mars^18^, Paris meteorite^19^, dwarf planet-asteroid Ceres^20^, Grove Mountains meteorites, Antarctica^21^, Nogoya meteorite, Argentina^22^, Aguas Zarcas meteorite, Costa Rica^23^. Therefore, the geological conditions of cronstedtite formation should be specific.

Cronstedtite is less abundant and unstable Fe end-member variety of serpentine family in rocks, reflecting the replacement of cronstedtite by Fe–Mg serpentine as alteration progresses^24–28^. As Fe-rich components are used up, formation of Fe–cronstedtite ceases and phyllosilicate depositing directly from attack of anhydrous components is of the more Mg-rich variety^28^. The formation of cronstedtite may indicate the composition of the original olivine rich in iron. At the same time, the conversion temperature of high-iron olivine should be extremely low, since serpentinization of this mineral in deep conditions is apparently impossible. Cronstedtite forms at more oxidizing conditions (lower *f*H_2_) and lower activities of dissolved silica than greenalite (Fe–serpentine)^29^. Cronstedtite forms at more reducing conditions than magnetite and goethite. The Mg-rich cronstedtite could be stable at slightly higher *f*H_2_ and aSiO_2_(aq) values than Fe-cronstedtite. Thus, low-P and low-*f*H_2_ conditions needed for formation of cronstedtite in processes of hydration of anhydrous Fe–Mg silicates^29^. As altered product of the Fe–Mg silicates cronstedtite may reflect specific conditions of transformation primary minerals. In this sense, we can estimate temperatures, log*f*O2, pH, water/rock (W/R) mass ratio et al., since these parameters have been studied in detail for cronstedtite stability conditions^26,27,29–34^.

A detailed description of the cronstendtite formation conditions in massive sulfide deposits is very limited in the public literature^35^, unlike, for example, extraterrestrial bodies, especially in carbonaceous chondrites (CM)^22,24,25,27–29,31,33^, in which it is the most common, highly sensitive to altered conditions, and constantly occurring mineral.

Iron is poorly preserved in clay minerals and is displaced by iron oxides and hydroxides, or enters the structure of kaolin in very small quantities^36^. However, there are also rare representatives of such minerals as berthierine, greenalite, and cronstedtite is one of the most ferruginous varieties among others.

Cronstedtite belongs to the kaolinite–serpentine group^15^. It is an Fe-rich phyllosilicate comprised of a tetrahedral (T) and an octahedral (O) sheet (T–O or 1:1 layer). The general formula of cronstedtite is (^II^Fe_3*−x*_^III^Fe_*x*_)(Si_2*−x*_^III^Fe_*x*_)_2_O_5_)(OH)_4_ (where 0 < x < 1). Fe^3+^ is present in the tetrahedral sheet and this allows for discriminating cronstedtite from other Fe-bearing phyllosilicates. Several polytypes are known, such as 1M polytype (space group Cm, crystal system monoclinic), 3T (space group P31, crystal system trigonal) belong to subfamily A^37^. Both polytypes occur separately or in mixed^13^. 1T (space group P31m, crystal system trigonal) polytype belongs to subfamily C^17^. 2H2 polytype (space group P63, crystal system hexagonal) belonging to subfamily D serpentine^38,39^. Recently, a new non-MDO polytype, 6T2, was discovered in Pohled, Czech Republic^40^.

Cronstedtite crystals exhibit a variety of forms: triangularly, tabular or pyramidal, conical, acicular, columnar, cylindrical, as well as sheaf- or barrel-like. They always have a perfect cleavage along the basal plane parallel to the structure layers and perpendicular to the stacking direction. The color is black, but the thinnest cleaved plates are dark reddish in transmitted light^14^.

This paper presents new data of the first findings of cronstedtite which formed via the alteration of Fe–Mg-bearing primary silicates^22^. The cronstedtite sporadically contains impurities of Al, Ca, and Ni, and is spatially associated with Cu–Fe–Ni sulfides, magnetite, and platinum-group elements (PGE), particularly Pd-rich phases. According the X-ray diffraction analysis for the bulk massive ores besides cronstendtite are established kaolinite, gypsum, calcite, quartz, and cristobalite.

## Geological background

**Figure 1:**
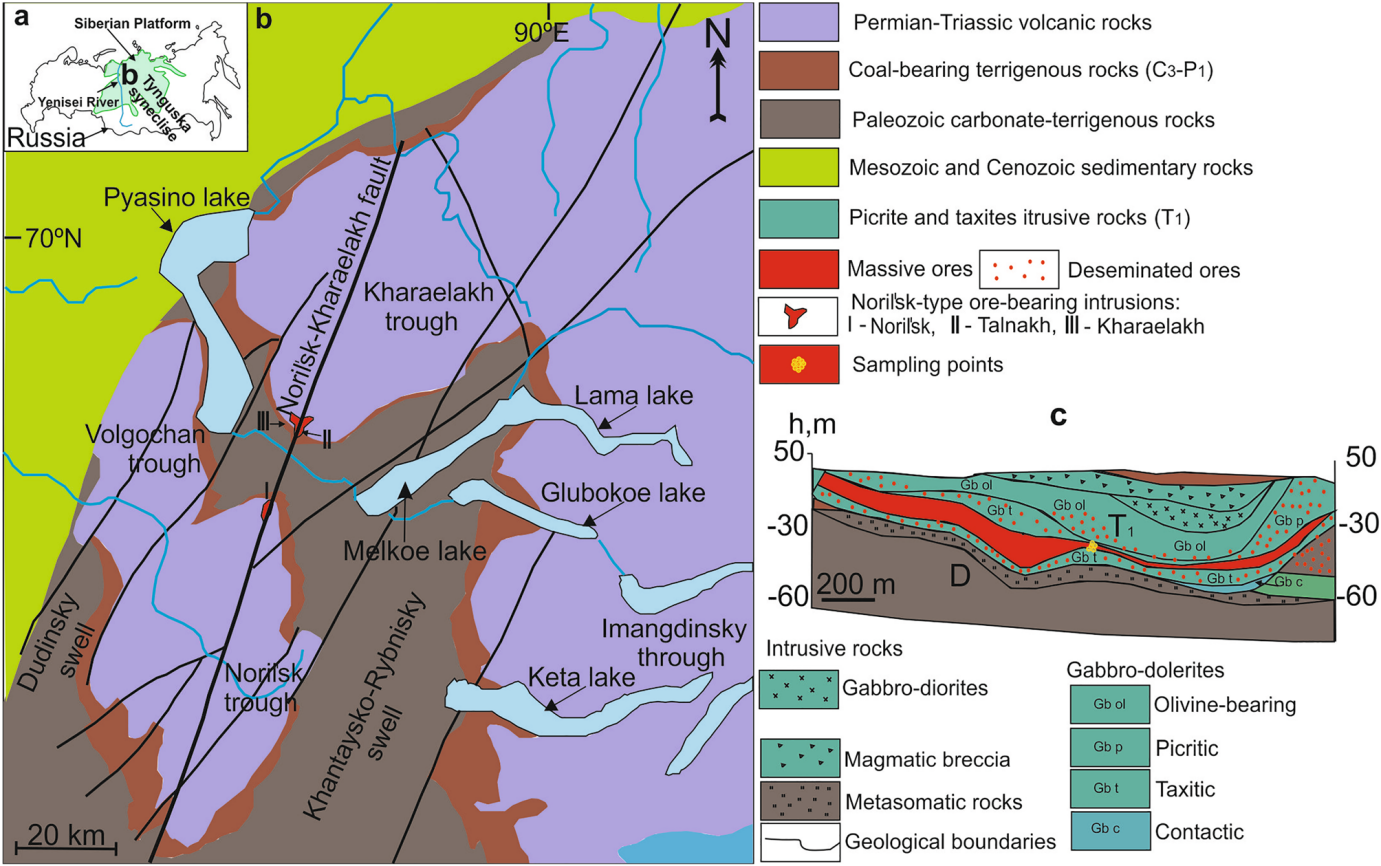
Location of the mapped area in Russia (**a**); simplified geologic map of the Noril’sk area (modified after^45^) (**b**) and cross-section through the Southern-2 orebody of the Talnakh intrusion (**c**) modified from Ltd. Noril’sk Geology materials.

The Noril’sk area is located within the Arctic Circle, on the right bank of the Yenisei River (Fig. 1a). Tectonically, it consists of several tectonic structures including Kharaelakh and Noril’sk troughs, Vologochan trough, Dudinsky and Khantasko-Rybninsky swells and western part of the Tunguska syneclise (Fig. 1b). The eastern boundary of the Noril’sk region coincides with the western part of the Tunguska syncline, located on the Siberian platform. This area includes Cambrian–Permian terrigenous sedimentary rocks and overlying volcanic rocks of the Siberian Traps^20,41,42^. Volcanogenic formations represent a clearly stratified stratum composed of basaltic covers with a thickness of a few meters to 90–100 m and horizons of tuffaceous rocks with a thickness of several tens of centimeters to 20–40 m. The ranges of such units vary in size, but sometimes they amount to tens of thousands of square kilometers, capturing the entire Nori’lsk area or going beyond it. The sequence is divided from the bottom to the top into the following formations: Ivakinsky (Late Permian), Syvermsky, Gudchikhsky, Khakanchansky, Tuklonsky, Nadezhdinsky, Morongovsky, Mokulaevsky, Kharaelakhsky, Kumginsky, Samoyedsky (Early Triassic)^43^. Lower part of the volcanic sequence consists of small high-Ti subalkaline and picritic flows (3.8 wt% Ti) while upper part includes low-Ti thick tholeiitic flows (1–1.5 wt% Ti)^42,44^.

Numerous mafic intrusions, among which the most economically productive in terms of Cu–Ni–PGE reserves—Noril’sk I, Talnakh, Kharaelakh formed during the Early Mesozoic, also assigned to the Siberian Traps, intruded the sedimentary rocks under the volcanic pile. Intrusions form bodies similar to ribbons. They are long and narrow.

The Talnakh intrusion lies at the boundary of the coal-bearing terrigenous rocks of Tunguska series (C_3_-P_1_) and Paleozoic carbonate-terrigenous rocks in a highly fractured block of rocks (Fig. 1c). In the vertical section, they are represented (from the bottom to the top) by taxitic and picritic gabbro-dolerites, which occupy ~ 50% of the section, and significantly less gabbro-diorites (Fig. 1c). Sulfide ores occur as droplets and aggregates in picritic and taxitic gabbro-dolerites and also form a vein of massive ores within the intrusive body (Southern-2 orebody)^6^. Samples containing cronstedtite were taken from the massive ores indicated on the schematic map (Fig. 1c).

## Results

### Rock texture and mineralogical features of the cronstedtite-bearing samples

**Figure 2:**
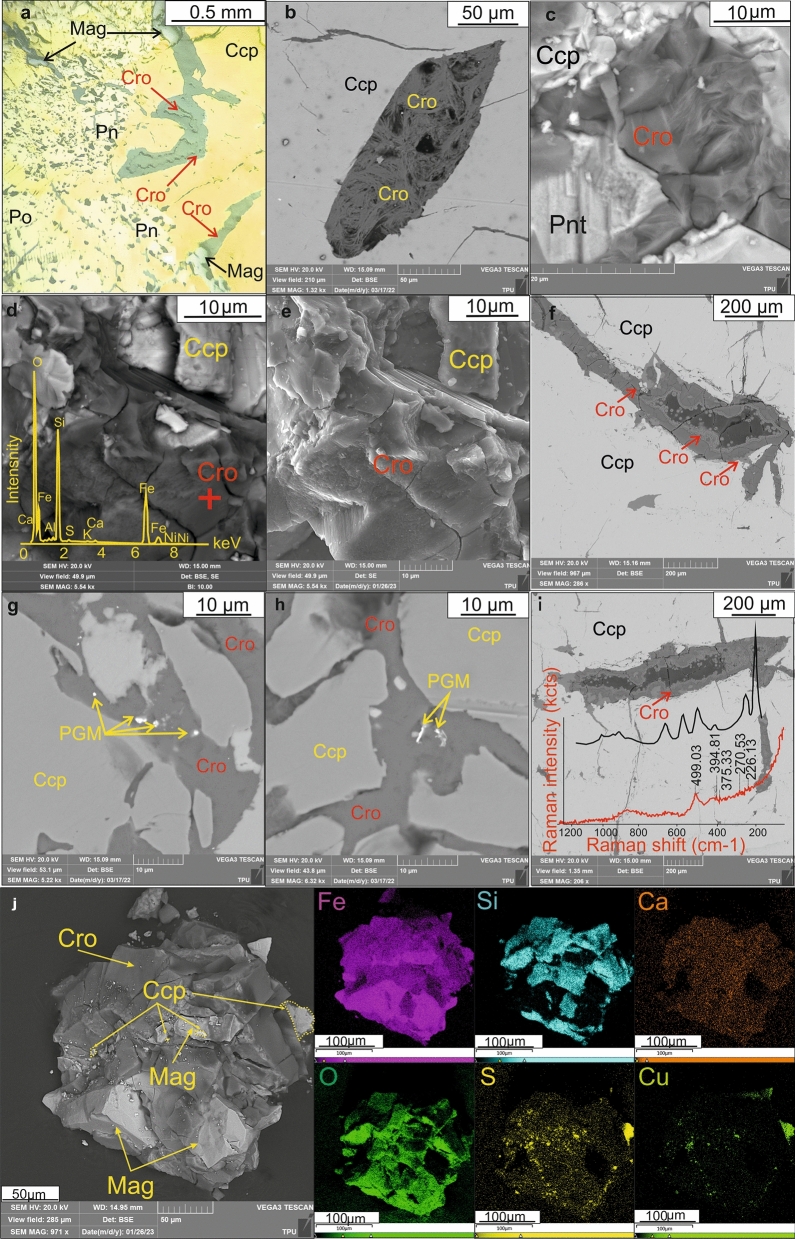
Transmitted-light (**a**), backscattered (**b,c,d,f,g–j**), secondary (**e**) electron photomicrographs, and elemental map (**j**) illustrating of cronstedtite (Cro) intergrown and intersected with magnetite (Mag), chalcopyrite (Ccp), pentlandite (Pn) and pyrrotite (Po) forming massive ores (**a**); cronstedtite (Cro) with a radially radiant (**b**), fibrous (**c**), and well-marked layered structure (**d,e**), filling the cracks (**f–i**) in association with PGM (**g,h**); (**i**) Red line showed analyzed Raman-spectra of studied cronstedtite while black line corresponds to Raman-spectra of cronstedtite 3T polytype from Kisbanya, Hungary^36^. (**j**) The joint association of magnetite and cronstedtite and the maps illustrating the distribution elements on this association.

The samples used for the study represented by massive sulfide ores from the Southern-2 orebody of the Talnakh intrusion^6^. They are mainly composed of copper (chalcopyrite) and iron (pyrrhotite, pentlandite, troilite) sulfides. A variety of PGMs occur in these rocks as well^11^. The sulfides in the orebody are crosscut by dark veinlets composed of oxides and silicates, the total amount of which does not exceed 1–3 vol% of the sample^11^ (Fig. 2a). The main silicate within the vein is an Fe-rich silicate that occurs as radiating, fibrous aggregates (Fig. 2b,c). In secondary electron images, the layered structure of this Fe-rich silicate is readily visible (Fig. 2d,e). Using an EDX detector of Scanning electron microscope, and data from TEM impurities of Al, Ni, Ca were detected (Figs. 2d, 4b). In addition, we observed layer-by-layer growth and sequential filling of cracks between sulfides (Fig. 2f). In some places, this mineral contains micron-sized PGM (Fig. 2g,h). The Raman spectrum of this mineral demonstrates certain differences from the standard item of cronstedtite from Hungary^36^ (Fig. 2i). The presence of impurities Al, Ni, Ca can affect and distort the pattern of the spectrum. The joint association of magnetite and cronstedtite and the maps of the elemental composition of this mineral association are shown in Fig. 2j.

### Powder XRD and Infrared characteristics

**Figure 3:**
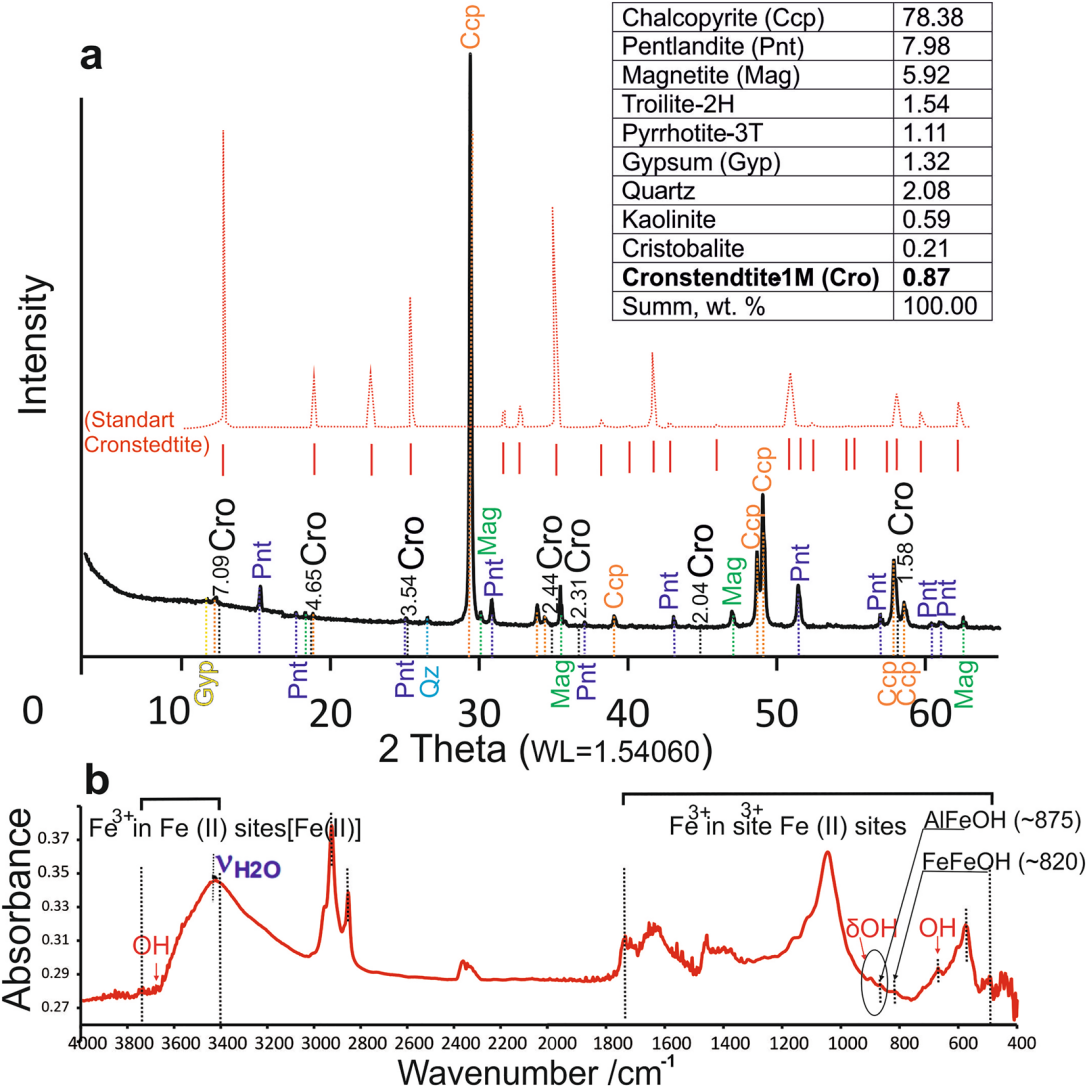
XRD pattern based on balk composition of cronstedtite-bearing massive sulfide ores (**a**) and Infrared (IR) spectra of monofraction cronstedtite (**b**) from massive Cu–Ni–Pt–Pd sulfide ores in Talnakh intrusion (Noril’sk camp). (**a**) Red line showed XRD pattern for pure standart cronstedtite crystal using the structure from Ref.^36^.

The X-ray diffraction (XRD) pattern for the bulk massive ores is presented in Fig. 3a with comparison a spectrum for pure cronstedtite (red line) from Ref.^36^. The reflects at 7.09, 4.65, 3.54, 2.44, 2.31, 2.04 and 1.58 Å are characteristic of cronstedtite. Our powder diffraction data roughly corresponds to the simulated patterns of 1M or 3T polytypes, or their mixed. Both polytypes belong to the Bailey’s group A and provide identical powder diffraction patterns^37^. Other mineral phases that occur with the cronstedtite are kaolinite, gypsum, calcite, quartz, and cristobalite (Fig. 3a). The infrared (IR) spectrum reflecting the composition of cronstedtite monofraction is shown in Fig. 3b. The IR data is represented by specific information reflecting the vibrational spectroscopy of mineral, and using as a complementary method to X-ray diffraction (XRD)^46^. Received spectrum provide information on structural OH^−^ groups and H_2_O in clay mineral^47^. The area near 3400 cm^−1^ corresponds to the complex water band (H_2_O group), while the presence of the OH^−^ group reflect area ~ 900 cm^−1^. Diffuse reflectance IR spectra in the 700–1200 cm^−1^ variable amounts of substituted Fe^3+^ in Fe(II) sites^48^. Diffuse reflectance IR spectra in the 3400–3750 cm^−1^ containing variable amounts of substituted Fe^3+^ in Fe(II) sites ([Fe(II)])^48^ (Fig. 3b).

### TEM description

**Figure 4:**
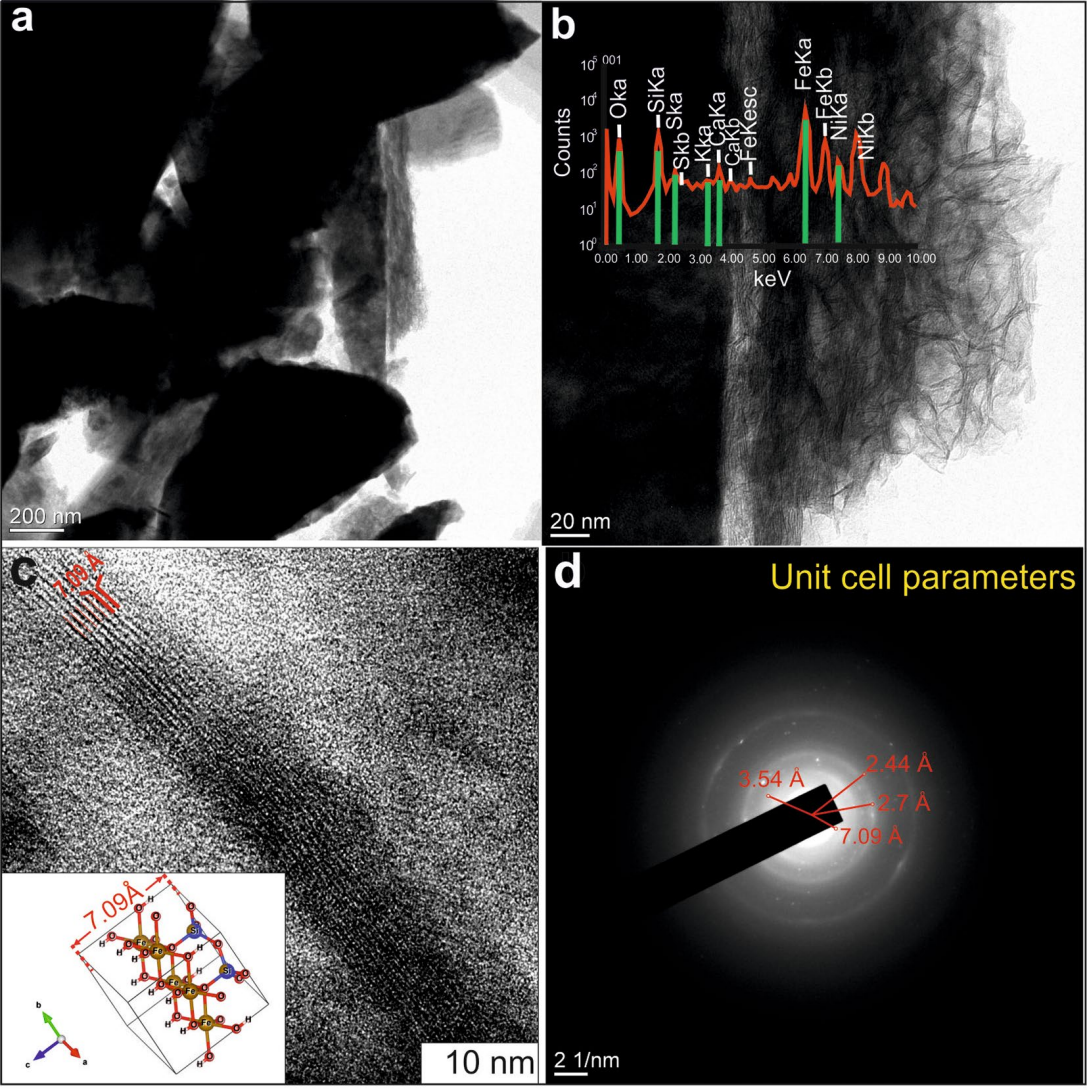
TEM images of cronstedtite demonstrating triangular (**a**) and fibrous (**b**) crystals; linear interplanar distances between the basal planes of tetra- and octahedral grids (**c**), and local electron diffraction pattern (**d**).

The shape of analyzed under transmission electron microscopy (TEM) crystals—triangular (Fig. 4a) and fibrous (Fig. 4b), in some TEM images seems to be characteristic for the Bailey’s group A, possible polytypes 1M, 3T or 1T belongs to subfamily C^37^ according to their crystal habit, unit cell parameters, and powder diffraction data: 7.09, 4.65, 3.54, 2.44, 2.31, 2.04, 1.58 Ang. The linear interplanar distance between the basal planes of the tetra- and octahedral planes is 7.09 Å (Fig. 4c), which corresponds to *c* lattice parameter of cronstedtite. This is confirmed by the local electron diffraction pattern (Fig. 4d), as well as by the lattice parameters 3.54 and 2.44 Å. According to the parameters of the unit sell, cronstedtite is more corresponds to trigonal space group P31m.

## Discussion

The petrographic features observed by SEM equipped with EDX for the polished sections of massive sulfide Cu–Ni–PGE ores from the Southern-2 orebody of the Talnakh intrusion (Noril’sk area) indicated occurrence of fine-grained, fibrous needle like Fe-phyllosilicates clusters < 50-µm-sized grains. According the X-ray diffraction analysis of the bulk massive Cu–Ni–PGE ores, transmission electron microscopy, scanning electron microscopy, Raman and Infrared spectroscopy of clay monofraction the cronstendtite (7.09 Å along its *c*-axis) was established, but further identification of the specific polytype is hampered by the small size of the crystals. The cronstedtite from the Southern-2 orebody sporadically contains signals of Al, Ca, and Ni. It is possible that Ni signal came from the adjacent sulfide minerals, but Al and Ca impurities probably occupies a portion of the structural sites that are traditionally filled by Fe^3+^^49^. Despite decades of mineralogical study of the massive sulfide Noril’sk ores, the structural identity and diversity of clay and other secondary minerals in these rocks is only partially resolved^50–53^. Cronstedtite is a major constituent of CM chondrites, but the conditions under which it formed are still largely underconstrained due to their scarcity in terrestrial environments^33,34,54^. The difficulty to quantify the secondary minerals of CM chondrites is related to many factors: (i) direct samples of alteration fluids are extremely rare and difficult to analyze, (ii) the setting(s) of alteration remains controversial, (iii) fine-grained mixtures leading to analytical problems for their characterization and (iv) the stability field of secondary minerals are largely under-constrained because of their scarcity on Earth^54^. In this regard, each a new finding of cronstedtite in a terrestrial environment deserves special attention.

Cronstedtite abundance depends on salinity, pH, carbon concentration and time-integrated flux of aqueous fluid. Moreover, there are abundant water, implying fluid flow, and a significant reservoir of oxidized carbon are required for cronstedtite formation^31^. According to Ref.^26^ the Fe/Si ratio decreases with increasing degree of alteration, which suggested resulted from the formation of phyllosilicate phases containing higher Mg/Fe ratios^55^. Thus, the content of cronstedtite in rocks decreases with increasing altered changes.

Laboratory experiments and thermodynamic calculations suggest that cronstedtite forms under following conditions (log*f*O_2_ ~ − 75 to − 55) in neutral conditions (pH 7–8) from Si- and Fe-rich fluids at 50–120 °C^29,30,32^. The inferences about formation of cronstedtite at low *f*H_2_ = − ∼10^−6^ to 10^−1^ and water/rock (W/R) mass ratio = ∼ 10^7^ to ∼ 5^29^ at early stages of alteration anhydrous Fe–Mg silicates or in low-P open system environments are consistent by Ref.^56^. Cronstedtite forms at more reducing conditions than magnetite, and in alteration processes magnetite forms together with cronstedtite and after it^28^. According to Ref.^29^ the occurrence of cronstedtite may reflect turbulent and disequilibrium environments and suggests formation of cronstedtite at the early stage of alteration Fe–Mg silicates. According to Ref.^31^ increased CO_2_ content in the fluid has implications for the production of cronstedtite, lowering the range of water-rock ratios at which the phase is a dominant alteration product. Cronstedtite could also form from near-surface fluid generated by impacts^29^. Thus, according to the modern model of sulfide deposition in the Talnakh deposit the catastrophic vapor phase exsolution associated with a drop-in magma overpressure at the transition from vertical to horizontal magma flow enabled explosive propagation of chonoliths^57^. In this sense, Talnakh chonolith emplacement in the specific geological conditions (< 2 km below the Paleozoic land), and the active participation of the near-surface fluids may play an important role in the formation of cronstedtite in massive ores of the deep levels of the Talnakh intrusion. Talnakh ore deposit is unusually shallow depth for this class of ore deposit, compared with typical depth ranges of 3–25 km^5^.

The scarcity of cronstendtite in terrestrial environments, whereas serpentine with predominant Mg component is abundant, and the instability of cronstendtite, allows us to conclude, that isolated cavities within fresh pyrrotite–pentlandite–chalcopyrite massive ores of the deep levels of the Talnakh intrusion could be a favorable environment for the formation of cronstendtite similarly to ferrotochilinite from the Oktyabr’sky deposit^53^.

## Methods

Twenty polished sections of massive sulfide ores from the Southern-2 orebody in the Talnakh Intrusion were studied by electronic microscopy. A subset of ten polished sections were selected for more study by TESCAN VEGA 3 SBU scanning electron microscope (SEM) and an OXFORD X-Max 50 energy-dispersive adapter (EDS) operated at an accelerating voltage of 20 kV, specimen current of 12 nA, and a spot diameter of approximately 2 µm. The EDS detector was calibrated using Co standard.

Mineralogy of the crosscutting veinlets was determined using a Bruker D2 Phase X-ray diffractometer (Billerica, MA, USA) using Cu-Kα radiation at a current of 10 mA and a voltage of 30 kV. A fraction of the bulk-rock powders with <10 μm grain size was scanned from 8° to 70° 2θ, with a step size of 0.02°, scanning rate of 1.5 s, divergence slit of 1 mm, anti-scatter slit of 3 mm, and receiving slit of 0.3 mm.

Raman spectroscopy was performed using a Thermo Fisher Scientific DXR2 confocal Raman spectrometer. All measurements were carried out at a laser wavelength of 785 nm and a laser power of 20–25 mW. Spectra typically represent the average three accumulations, each acquired over 5 seconds over the wavelength range of 0–1200 cm^–1^.

IR spectroscopy was performed using a Shimadzu IR Prestige-21 IR-Fourier spectrometer in the absorption mode and in the range of 400–4000 cm^–1^ with a resolution of 2 cm^–1^.

## Data Availability

The datasets generated and/or analyzed during the current study are available from the corresponding authors upon request.

## Code availability

The code to reproduce the presented results is available from the corresponding authors upon request.
